# Neo-Darwinists and Neo-Aristotelians: how to talk about natural purpose

**DOI:** 10.1007/s40656-016-0123-0

**Published:** 2016-11-24

**Authors:** Peter Woodford

**Affiliations:** Faculty of Zoology, University of Cambridge, Downing St., Cambridge, Cambridgeshire CB2 3EA UK

**Keywords:** Teleology, Neo-Darwinism, Neo-Aristotelianism, Purpose of reproduction, Ultimate causation

## Abstract

This paper examines the points of disagreement between Neo-Darwinian and recent Neo-Aristotelian discussions of the status of purposive language in biology. I discuss recent Neo-Darwinian “evolutionary” treatments and distinguish three ways to deal with the philosophical status of teleological language of purpose: teleological error theory, methodological teleology, and Darwinian teleological realism. I then show how “non-evolutionary” Neo-Aristotelian approaches in the work of Michael Thompson and Philippa Foot differ from these by offering a view of purposiveness grounded in life-cycle patterns, rather than in long-term evolutionary processes or natural selection. Finally, I argue that the crucial difference between Neo-Darwinian and Neo-Aristotelian approaches regards the question of whether or not reproduction deserves the status of an “ultimate” aim of organisms. I offer reasons to reject the concept of an “ultimate” aim in evolutionary biology and to reject the notion that reproduction serves a purpose. I argue that evolutionary biology is not in the position to determine what the “ultimate” explanation of natural purpose is.

## Introduction

Why do birds migrate? The obvious answer to this question might appear to be that they do so in order to find food, a more favorable habitat, and favorable weather conditions that will allow them to survive and satisfy their needs. Yet this answer appears to many objectionable, or at least requiring serious technical qualification, because it implies a kind of goal or purpose that a bird has when it acts, and, more importantly, that this goal both describes and explains its behavior. Descriptions that impute goal-directedness to activities and processes in nature are called *teleological* and the question of whether, and in what sense, this language of function and purpose can be valid in the study of life and its evolution is one of the perennial problems in philosophy of biology.

Ernst Mayr’s classic 1961 article in *Science,* “Cause and Effect in Biology,” defended a now widely recognized approach to such teleological language by introducing the term “teleonomy” and claiming that there are two sorts of questions that evolutionary biology is in the position to answer when it comes to basic questions like why birds migrate: “How?” questions and “Why?” questions (Mayr 1961). These two questions established a distinction between so-called “proximate” causes that investigate the mechanics of “how” bird movement works and “ultimate” causes that tell us the reason “why” birds migrate. Mayr’s influential distinction was intended to outline how Neo-Darwinian evolutionary biology could domesticate the language of purposiveness on a solid scientific foundation without any spooky metaphysical remainder (Mayr 1961).

The first aim of this paper is to examine more carefully how Mayr and Neo-Darwinian evolutionary biologists since Mayr have dealt with the status of teleological language and to provide a typology for organizing the philosophical options that are currently available. The second aim is to juxtapose these “evolutionary” approaches with alternative “non-evolutionary” approaches to purposiveness in biology recently developed in writings of the Neo-Aristotelian philosophers Michael Thompson and Philippa Foot (Thompson 2008; Foot 2003; Grene 1974). In the course of setting out these approaches, I argue that the key concept for “non-evolutionary” approaches is the concept of a life-cycle pattern. In the final section, I argue that differences between these two approaches to the theoretical foundations of teleological language reveal a deep disagreement over what sorts of answers can be given to “why” questions in biology. I contrast these approaches by analyzing how each would address the question of the purpose or function of reproduction and argue that there is a flaw in Neo-Darwinian assumptions about the purpose that reproduction serves. By criticizing both the notion that reproduction serves a purpose and the notion that reproduction should have the status of “ultimacy” with regard to an organism’s activities, I show that non-evolutionary approaches offer a desirable corrective to the idea that evolutionary biology offers an “ultimate” explanation of life and purposiveness in nature.

## Are there natural purposes?

It appears obvious to the many “lay” viewers of David Attenborough documentaries, thanks to their evocative narrator, that the arena of life requires concepts of challenge, struggle, success, achievement, and strategy to be understood. Organisms face ecological challenges that they must overcome; life itself is a challenge, a struggle, and even a competitive game. However, the use of language of avoiding costs to reap benefits, the application of optimality models, and the use of game-theory to understand evolutionary strategies and pay-offs show that intentional language is not only restricted to the laity. Depicting an organism’s life as the overcoming of a series of challenges, as requiring ecological problem-solving, or conceiving behavior as competitive or cooperative implicitly relies on an intentional-sounding language of implied objectives whose place in nature can seem puzzling. A challenge is something that stands in the way of a goal, a strategy is a plan of action toward a goal, and cooperation or competition usually only ensues between two or more parties striving to achieve the same objective. It appears hard to shake off the sense that the metaphor of a game really does capture something about life and its evolution, and that the producers of these documentaries have done an extraordinary job acquainting us with astonishing strategies within it.

The philosophical puzzles that this language raises are basic and they address our understanding of basic properties of the physical world: can blind bits of matter *really* have purposes? Can living things have goals and objectives, can they really be said to be competing or even cooperating when they have no knowledge of these goals and no idea of the eventual effects of their activities? Is such language just an anthropomorphic or metaphorical projection, or perhaps a pragmatically useful heuristic, or does the living world *really* present an arena that contains purposive organization and directed activity? The problem of teleology in nature has been a locus of philosophical interest since Aristotle because it was at the basis of his understanding of life, nature, and of the organization of the cosmos as a whole. It is particularly important because Aristotle regarded human capacities of theoretical and practical reasoning, goal-directed agency, and intention as instances of more general purposive features of the biological world. Many philosophers today still find it compelling to distinguish living things from non-living things within the realm of different types of entities specifically through their apparently purposive organization and behavior and through the resistance that this organization poses to being explained away or reduced to principles of the non-living matter that form its constituent parts. This paper is concerned with the purposive characteristics of living activity generally, and not with the more obvious purposive nature of conscious human behavior and thought, although this might be seen as a local example of the more general problem.

It is clear why it appears that these philosophical puzzles must be addressed today by reflecting on the most powerful explanation of the organization of the living world and its change over time that we possess: Darwin’s theory of evolution by natural selection. Pioneers in Neo-Darwinian evolutionary biology like R.A. Fisher, J.B.S. Haldane, William Hamilton, Richard Lewontin, and John Maynard Smith established the rigorous theoretical foundations that now underwrite widespread notions of games, strategies, and success. Works like Richard Dawkins’ *The Selfish Gene* in 1976 further cemented notions of evolutionary winning and losing in scientific and lay discourse. The natural way of speaking about living things as navigating ecological challenges, solving problems, having interests, competing and cooperating to gain benefits and avoid costs is part of conceptual armory of both scientists and the popular audience. Yet this language still generates puzzles because of the directed character of the activities it describes.

Ernst Mayr’s early 1961 paper was a classic attempt to delineate a sound theoretical and scientific basis for the teleological notions of purposiveness that underlie these strategic conceptions of the living world within a structure of explanation focused exclusively on the question of causation. Mayr divided the central questions of biology first into “How?” questions, which seek the physiological and eventually genetic mechanisms that underlie the various capacities, functions, and behaviors of living things. But, Mayr also argued that ultimate questions of “Why?” fall within the purview of evolutionary theory and these were the questions gave rise to teleological language. Our original example can help distinguish what we might be looking for in asking these two kinds of questions. We might ask the “how” question of what *causal* mechanisms trigger bird flight, sustain it, and are involved in ending travel, but we might also ask “why” a bird migrates by seeking a r*ationale* for this behavior and treating the bird as if it were seeking to achieve certain aims and acting strategically to do so.

Understanding how Mayr approached the “Why?” question is crucial for understanding how he was able to solve, or perhaps better dissolve, the puzzle of teleological language. For Mayr, when we ask “Why?” an animal is the way it is or does what it does, the answer is really a re-packaged sort of “How?” question. Mayr’s “Why?” does not really ask *for a reason,* for a *purpose*, or for a *rationale*, but rather for another sort of causal mechanism that is not to be found within the lifespan or current functioning of the individual organism, but in its phylogenetic ancestry and evolutionary history (Mayr 1961, p. 1503). The reason “why” the warbler on Mayr’s New Hampshire porch started his southward migration on the summer night of August 25th, he famously argued, is because their ancestors who showed the same heritable behaviour reproduced successfully. No warbler needs to be understood as having any goal—most importantly, it need not even be conceived as having the goal of fitness or successful reproduction to account for any of its individual behaviors, such as migrating. The warbler’s purposive-seeming behavior is just the playing out of a genetic “program,” as Mayr called termed it (Mayr 1961, p. 1504). This behavior was the effect of an ordered mass of evolved proximate causal mechanisms that we find in nature because they were the products of a real causal series of successful reproducers. For Mayr, where there is no intention or representation of the action, there is no real purpose or rationale.

So, Mayr really erased any valid notions of objectives from the biological world by reformulating the implied “in order to…” that is used to describe the act of travel that the warbler undertakes as another type of mechanistic and causal “how?” question. This offered a strategy for seriously qualifying, denying, or rendering unnecessary teleological rationales that is still common among working biologists describing and seeking explanations of the behaviors and traits of living things. But not all evolutionary biologists or philosophers deal with purposive language in this manner. Niko Tinbergen’s famous “four questions” provides an analytic framework of evolutionary thinking that has been especially influential for scientists studying behavior and that preserves a more robust role for notions of purpose (Tinbergen 1963). Alongside problems of ontogeny and phylogeny that Mayr used to distinguish between so-called “proximate” and “ultimate” causes, Tinbergen included the question of adaptive “function” as a fundamental biological *explanandum.* Tinbergen accepted that the function or purpose of a trait is a genuine phenomenon to be explained, independent of proximate or ultimate causal mechanisms, but the adjective “adaptive” ensured that functions were to be explained solely in terms of non-purposive effects on survival and reproduction.

Indeed, the field of behavioral ecology today views behavior as a “trait” like any other that is subject to natural selection and follows Tinbergen in conceiving of behavior too in functional terms to be explained through the effect of behavior on reproductive success (Krebs, Davies, and West 2012). What is important to notice here is that Tinbergen’s more robust concept of adaptive function underscores a new kind of biological cause, another answer to a “why” question that begins “because…”, in addition to Mayr’s two conceptions of biological causation. Mayr’s warbler migrates south on August 25th *because* the behavior serves an adaptive purpose related to survival and reproduction. In this usage, teleological language does capture something about biological reality that cannot be captured without it or rendered in non-teleological terms, but this teleological character is still “ultimately” explained by the features of how the evolutionary process of natural selection works.

For readers familiar with the large philosophical literature on functions, my goal in the following is not to rehash important debates over etiological and dispositional accounts of function (See McLaughlin 2001 for an excellent summary of these debates and for defense of an Aristotelian position; see also Ariew, Cummins, & Perlman 2002 for a wide-ranging set of recent essays; and Lewens 2005 for a detailed and informative discussion of teleological language in biology). Instead, my aim is to relate these debates to what we might call “meta-theoretical” disagreements related to teleological language. This initial discussion of Mayr and Tinbergen allows us to make a few distinctions regarding the ways evolutionary biologists can interpret purposive language, its basis in nature, and its validity. Both Mayr’s and Tinbergen’s reformulations show subtly distinct paths. One path is to argue that organisms, their traits, and their behaviors are not purposively organized or directed at all, and that this is merely an anthropomorphic human error that gives a false picture of nature and is to be avoided. This can be called *error theory of teleology*, in analogy to the “error theory” in moral philosophy which claims that there are no mind-independent normative facts that our moral judgments are guided by. Mayr’s reformulation of “why” questions into “how” questions of biological programming and both genetic and evolutionary causation can be read as the view that teleological judgments are systematically false and misleading. When we ask the natural question of “why” an individual organism does something, the only legitimate answer refers to physiological causes and evolved mechanisms, not to rationales, goals, or purposes. This discussion of biological teleology of course sets aside for the moment the peculiar case of organisms with higher cognitive capacities, such as ourselves, whose intentional capacities may resist such an explaining away of its directed features.

The second strategy is to argue that although teleological judgments are anthropomorphic, the idea of a purpose or objective has heuristic value and it is helpful—indeed necessary—to view living things “as if” they are purposively organized and directed, even if they are not in fact so. This view can be called *methodological teleology*. It has its historical roots in Immanuel Kant’s *Critique of Judgment*, which argued that teleological judgments ascribing purposiveness to organisms and their traits are regulative principles that guide our inquiry into nature rather than constitutive principles that make experience of a mind-independent world possible. Contemporary evolutionary thinkers often modify this Kantian doctrine by proposing that teleological language can be useful as a shorthand for more technical and scientifically correct causal descriptions, like Mayr’s, that are too cumbersome to use on every occasion. Daniel Dennett’s “design stances” is an instance of such a view (Dennett 1987, p. 16). We talk of organisms making decisions, executing strategies, and having wants, needs and drives, and seeking reproductive success, but this is a deliberate shorthand for enormously complex mechanical processes that make up organisms and the causal history of mutation and selection that has generated them. The mechanisms underlying observed features of biological entities may be thought to be too complex for the human mind to grasp comprehensively (at present or perhaps permanently) and so we might require such simplifying shorthands. Alternatively, such teleological heuristics can be thought of as satisfying our evolved intuitive cognitive capacities, such as an innate “theory of mind,” which has itself been fashioned by the selective evolutionary pressures of complex social environments to detect intentionality and seek “naïve” biological explanation in terms of rationales and purposes (Atran 1998). These innate capacities account for the cognitive need we have to explain traits and behaviors according to *purposes* and *reasons* in addition to identifying *causes* and *mechanisms* that gave rise to them (See Godfrey-Smith 2009 for a critique of evolutionary explanations in the form of rationales). In either of these two strategies, teleological judgments concerning the purposive activity and organization of living things may be a useful aid, even to scientists, but they are not, strictly speaking, true. *Methodological teleology* should, then, be regarded as a special form of *error theory* because its account of teleology maintains an anti-realist stance, and I submit that it is probably the dominant view in the philosophy of biology today.

Mayr’s view is a form of *error theory* and Tinbergen’s minimalist sense of “function” as adaptive effect on reproductive success can be neatly fitted within Mayr’s largely reductionist and genome-centered picture in the form of *methodological teleology*. But there remains a third Neo-Darwinian perspective on teleology that need not be anti-realist in these senses and that can defend a more robust version of Tinbergen’s notion of function. This view argues that organisms are *really* purposive in organization and behavior and that it is precisely Darwin’s theory of evolution by natural selection that licenses this teleological conception. This position can be called *Darwinian teleological realism*. Such a view sees Darwin’s achievement as two-fold. Not only did Darwin explain evolutionary change over time through differential effect of traits on reproductive success, he also provided a principle of natural, non-intentional, design in the living world (Gardner 2009; Grafen 2006; West and Gardner 2013). A view of this sort stresses that Darwin’s theory did not change the subject or eliminate the discourse of design found in the writings of natural theologians like William Paley, but rather replaced theological and super-naturalistic design-principles and purposes with design-principles grounded in an understanding of the causal structure of the evolutionary process and observation of the ecological conditions that organisms inhabit. Organisms are purposively organized wholes, their traits do serve purposes, they have goals, and these goals explain their behavior. Darwin’s view of the evolutionary process is to be even more admired because it explains both *how this is possible* and *what the goal of an organism really is*. The important point is that this conception is committed to a more robust, realist view of teleology and it sees evolutionary biology as licensing this.

## Neo-Darwinian purposes in biology

The *Darwinian teleological realist* conception has always revolved around the concept of “fitness” and the two distinct roles that this concept can and has played. First, “fitness” captures the Darwinian insight that traits that positively affect the number of reproducing offspring of their bearers will come to proliferate in populations of living things over time. It is a description of the mechanism by which populations change over time in ways that accumulate adaptive traits, which are simply traits that belong to successful reproducers and contribute to reproductive success. This notion of fitness is, at least theoretically, if not always practically, a measurable quantity that captures the underlying causal structure of evolutionary outcomes, especially the complex and local adaptations that astonished naturalists like Paley and Darwin and still astonish us. Biological traits and capacities initially arise as a result of the cumulative process of descent, inheritance, and modification, and they spread through populations only because they have a positive or neutral effect on reproductive success.

The second notion of “fitness” is a *telos*, or goal that accounts for the unified functioning and organization of the whole organism. It is a “design-principle” that explains how the parts of an organism fit together to form a functioning, unified whole by identifying a so-called “ultimate” objective that all traits and behaviors of an individual contribute to (Gardner 2009; West and Gardner 2013; Fitzpatrick 2011). Darwin’s account of adaptive change through natural selection provided a principle of what an adaptation *is*, namely, a trait that has been preserved and spread *because* it had the effect of increasing reproductive success of individuals relative to those without the trait. Thus, evolutionary biology answers the question of “why” traits have been selected by appealing to a cause that also offers a reason or rationale: because they contribute to reproductive success. Over the *long dureé* of evolutionary time, as organisms evolve and accumulate adaptations, all of their parts become more and more functionally integrated around the single, unified aim—the key evolutionary criterion of selection. Reproductive success then acquires the status of a principle of unity and organized functioning of the whole organism. It explains “why” an organism is organized the way it is and “why” it behaves the way that it does through reference to a goal. Rather than eschew evolutionary rationales like Mayr, the Darwinian teleological realist argues that the process of evolution by natural selection produces organisms that are designed to seek and achieve a goal.

Defenders of the idea of “fitness” as the “ultimate” goal of the organism argue that this conception can be derived through reflection on how the evolutionary process that generates organisms imprints itself upon them. Alan Grafen has written extensively in defense of mathematical models that depict evolution as a process of optimization and his work is a prime example of a defense of *Darwinian teleological realism*. He argues that this view has its roots in R.A. Fisher’s fundamental theorem of natural selection, which states that “non-random changes in gene frequency always change mean fitness positively. No other aspect of the genotypic changes of a population has a systematic optimizing tendency” (Grafen 2003, p. 325). He writes further that “the fundamental theorem tells us what it is that the design-creating capacity of evolution regards as good design. It must increase the partial fitness *of the individual*: not the individual’s longevity, or happiness, or intelligence or complexity” (Grafen 2003, p. 326 my emphasis; Fitzpatrick 2011). For Grafen, it is the optimizing tendency of evolution by natural selection that justifies the conception of a unified ultimate goal as the crucial explanatory principle in evolutionary theory. Because organisms behave as “rational creatures maximizing a utility function,” one can model behavior and speak of the purpose of an organism as a whole in relation to the ultimate, or final, end of reproductive success (Grafen 2003, p. 326).

Conceptions of fitness have of course been modified since Darwin, as shown by foundational debates in social evolution that have sharpened these issues. Since William Hamilton introduced the concept of “inclusive fitness” in 1964, researchers in the evolution of social behavior have challenged the idea that individual reproductive success *simpliciter* is the *telos* that explains organismal functioning and behavior (Hamilton 1964). Hamilton helped make sense of traits that proliferate and yet do not appear to increase the fitness of organisms that possess them. The idea of “indirect fitness” was used to understand the organization and behavior of organisms that are not capable of reproducing at all, like sterile worker ants, for whom it would for obvious reasons be problematic to claim that the natural goal for which they are organized is *their* individual reproductive success. Today, influential theorists, including Alan Grafen quoted earlier, regard Hamilton’s central insight to be that the aim of individual organisms is the spread of their genetically heritable traits, whether “directly” through their own reproduction or “indirectly” by aiding the reproduction of relatives who are highly likely to share those traits (Grafen 2006; West and Gardner 2013). This conception of “inclusive fitness” refines the second conception of “fitness” by identifying a subtly more expansive objective that accounts for the unified design of whole organisms, but it maintains the view of individual organisms as really goal-directed and agential in the sense I am outlining here (See Birch and Okasha 2015 for a helpful analysis of this issue).

Indeed, the very same defenders of the notion of an evolutionary design-principle argue that the notion of inclusive fitness helps us understand that groups of organisms can become purposive entities as well. Andy Gardner and Alan Grafen have argued that the same Darwinian logic that tells us what individual organisms are designed to do can predict circumstances under which social groups can and will become be adaptive entities as well. These are circumstances in which within group competition is negligible and the inclusive fitness “interests” (note that this widespread term also imputes teleological characteristics to organisms) of interacting individual organisms are aligned (Gardner and Grafen 2009). I will return to this point later on in considering the contrasts between Neo-Aristotelian and Neo-Darwinian conceptions of natural purpose. For now, these Darwinian teleological realists help us appreciate that theorists distinguish between a gene-centered focus on “units of selection” and a focus on whole organisms as “units of adaptation” where purposiveness begins to appear (Gardner 2014). Natural selection acts by changing gene-frequencies, but it is only at the level of whole organisms—and in special cases of purposively integrated groups (so-called “superorganisms”)—that adaptatively purposive traits appear.

This account of course entangles us in contentious theoretical debates currently going on in the science of social evolution. Mathematical biologists applying game theory to social evolution have recently questioned both “fitness” and “indirect fitness” as unified design-principles, asking whether evolutionary biology and the theory of social evolution require teleological principles to explain organismal behavior at all (Allen et al. 2013; Birch and Okasha 2015). Individual organisms, especially sterile ones, may be maximizing different fitness “quantities”—as mathematical biologists often refer to that which is maximized through behavior—in different biological systems and at different levels of biological organization: Genes, cells, individuals, and groups. Evolutionary biology should avoid the temptation to think in terms of universal quantities (such as number of offspring) that all organisms in general are seeking to maximize, and instead focus on what conditions lead to the outcome that certain behavioral traits, especially social ones that affect others of the same species, proliferate in dynamic populations. Ben Allen and Martin Nowak argue that “both theory and experiment have shown that frequency-dependent selection can lead to complex dynamical phenomena such as multiple and mixed equilibria, limit cycles, and chaotic attractors, ruling out the possibility of general maximands. Thus, evolution does not, in general, lead to the maximization of inclusive fitness *or any other quantity*” (Allen et al. 2013, p. 20138 my emphasis). These critics of the inclusive fitness theory of social behavior write further that a “straight-forward genetic approach” to the evolution of social behavior focuses on the question “under what conditions are mutations favored by natural selection? The target of selection is not the individual but the allele or the genomic ensemble” (Allen et al. 2013, p. 20138). It is ambiguous whether or not these critics of inclusive fitness reject the need for teleological principles altogether or if they simply reject any *general* or *universal* principles that apply to all biological systems as such. But there are philosophers who have taken these criticisms of all teleological “design-principles” to their limits. Peter Godfrey-Smith, for example, rejects the teleological and agential view of organisms in total, arguing that the only valid question for evolutionary biology is not “to what end?” a living thing acts or “who benefits?” from an organism’s traits but rather: “Suppose a population exists and some phenotype emerges—what is likely to happen to it?” (Godfrey-Smith 2009, p. 145).

These recent debates over the teleological character of the concept of inclusive fitness leave us with philosophical options that were already foreshadowed by Mayr. His approach is a historical antecedent of strategies that drop talk of goals and agency from biology altogether, concentrating on genetic programs and mechanisms while downplaying the explanatory significance or usefulness of recognizing whole organisms to be teleologically organized entities. This option is reinforced by the fact that individual organisms carry significance for the study of evolution primarily as temporary bearers of heritable traits, which are the “units of selection.” Since “straightforward genetic approach” to evolutionary change focuses on gene frequencies, the purposive organization of whole organisms and the directed character of their behavior need not come into view at all as a pressing *explanandum*, an *explanans*, or as a privileged “unit” of biological reality. *Evolutionary* biology is the study of the origins, spread, fates, and effects of these traits over time, and the attempt perhaps even to predict them; it can therefore ignore the scientific and explanatory significance of recognizing the agential character of whole organisms.

On the other hand, if the purposive organization and behavior of whole organisms is a genuine *explanandum*, the only valid evolutionary foundation that can account for it is the sort found in defenders of “inclusive fitness.” Defenders of the second conception of fitness uphold the “adaptationist” tradition that they rightly trace back to Darwin himself (See Birch and Okasha 2015; Lewens 2009 for helpful discussions of adaptationism). They underscore the view that Darwinism explains individuals and their life-cycles as purposively organized and acting wholes—as privileged “units” of adaptation—that come to exhibit this purposiveness precisely due to the dynamics of the process of evolution by natural selection. This process licenses the view of whole organisms as agents with real purposes, even if they are not aware of these purposes (Grafen 2003, p. 326). As we have seen, if the “goal” of reproductive success is a genuine one, a limited normative vocabulary of success and failure is licensed and so is a conception of what it means for an individual or population to “flourish” in evolutionary terms, namely to survive and reproduce over extended periods of time (Fitzpatrick 2011). Since individuals have an inherent Darwinian objective, anything that hinders the achievement of this objective is a genuine cost to the organism and anything that contributes to it is an evolutionary benefit.

This section has shown that there are disagreements about the status of teleological language internal to Neo-Darwinian theory and that Neo-Darwinian theory can align with either “realist” or “anti-realist” views of the teleological conceptions of the traits and behaviors of living things. The *error theorist* and *methodological teleologist* views reject the reality of teleological properties of organisms altogether, while the Darwinian “realist” sees the basis for such principles in the way that the selective dynamics of evolution shape organisms. This section has also aimed to show that regardless of which road one takes here, the problem of teleology is intertwined with the question of how we understand the scientifically valid ways of answering “why” questions. If the Neo-Darwinist has an answer to the question of “why” birds migrate that offers a *rationale* and refers to *real* “goals” or “purposes”—for example, of the sort that birds migrate *in order to* find insects to eat, etc.—these can only be the “ultimate” purposes of fitness or inclusive fitness maximization. A bird migrates in order to maximize its fitness or inclusive fitness; migration occurs *because* it contributes to survival and reproduction. In the next section, I consider an alternative, Neo-Aristotelian approach to the problem of teleology that maintains realism but rejects its Darwinian and evolutionary basis.

## Natural purpose for Neo-Aristotelians

Recent Neo-Aristotelian writers have developed an approach to purposiveness in biology independently of the Neo-Darwinian positions described above. These thinkers challenge sweeping statements like Theodosius Dobzhansky’s famous remark that “nothing in biology makes sense except in the light of evolution.” They argue that if organisms do require teleological concepts for what they *are* and what they *do* to be made intelligible, then the basis of this fact about what they are is independent of the question of how they have come to be in nature. Neo-Aristotelian philosophers Michael Thompson and Philippa Foot give purposive language a privileged place in descriptions of biological reality because they see such language as *constitutive* of our representations of this reality in the first place. Teleology is, for this reason, independent of and *prior to*—in the sense of constitutive of our representation of—biological entities, and so too of our inquiry into the Darwinian process through which their characteristics change over time.

The decisive differences between the Neo-Aristotelians and the previous positions we have surveyed can be seen already in their unique starting points. Thompson and Foot begin their investigation of the concepts needed to make sense of living things with a description of individual whole organisms, their activities within their wider ecological context, and the way particular behaviors and constitutive cellular and molecular processes fit into unfolding *life*-*cycle patterns*. As Thompson writes, teleological judgments do not offer a single, so-called “ultimate” objective that explains organism structure and behavior; instead, “they articulate relations of dependence among various elements and aspects and phases of a given kind of life” (Thompson 2008, p. 294). Thompson argues that even basic activities such as eating can only be identified *as eating* by regarding the animal’s locomotion, its chewing, the passage of the material through its digestive tract, its eventual defecation, etc. as processes of nourishment and waste removal. But by doing so, such events are already regarded as “vital activities,” that is, as events that have functional roles within the wider context of an unfolding form of life that make the future phases in the life-cycle possible (Thompson 2008, p. 57). To regard such phenomena *as* “vital activities” that contribute to the maintenance and development of the organism through its life-history is already to regard them as purposive and teleological. Moreover, teleological descriptions of “vital activities,” such as “eating,” identify principles of change and persistence in a living thing that are internal to its particular way of sustaining itself and making a living.

Thompson’s account of living individuals and life-cycle patterns is part of a more general and ambitious project. These vital descriptions of individuals, he argues, are possible only in relation to the conception of common, more general “life-forms” that individuals exemplify. The description of a shared “life-form” comprises what he calls a “natural history account” of a species or more general group to which individuals belong, and this account is constituted by a set of “Aristotelian categoricals,” as Thompson calls them. These are statements that are true of individuals but are also more true of the more general, supra-individual patterns of life that individuals are tokens of. The decisive point that Thompson makes is that our judgments about the functions, capacities, and behaviores of living things depend upon identifying such supra-individual patterns. An “Aristotelian categorical” would be of the form “warblers in New Hampshire migrate to the south at the end of summer.” Thompson’s key argument is that one can only *identify* and *describe* the warbler, its traits, and its behavior in the first place through “vital description.” To even call the warbler’s movement “migration,” or more fundamentally to call a chunk of DNA a “gene,” is to ascribe it a purposive place within a wider context of living activity (thanks to Micah Lott for this important point). It is crucial for the Neo-Aristotelian position that it is only on the basis of these teleological, vital descriptions that we are also able to judge whether individuals are functioning well or badly. For example, if a warbler failed to migrate and perished as a result, we would be in the position to judge that something had gone wrong in this particular warbler. The Neo-Aristotelian argues that this is not because we know from evolutionary theory that the warbler’s goal is to reproduce as abundantly as possible and it has not done so, but rather because we know from observing general patterns of warbler life-cycles that migration serves a vital function in allowing the warbler to sustain itself (Thompson 2008, p. 68).

Thompson’s treatment of biological teleology is merely preparatory ground-clearing for a larger argument that normative evaluation of human action is of the same logical form as judgments of the function and dysfunction of biological traits in living things more generally. These larger meta-ethical aspects of Thompson’s project require mentioning, but the salient point he makes about biological teleology for the purpose of this paper is that we can only first identify what any individual is doing “now” in terms of its vital activities, and we cannot arrive at an accurate conception of its vital activities except by relating them to more general life-cycle patterns that we have observed in other individuals who share the same life-form. Moreover, it is crucial to Thompson that judgments about life-cycle patterns are not merely statistical generalizations from individual observations, since most or all of the organisms we are observing need not live out their life-cycle and yet the “vital activities” that occur can still only be identified *as such* in relation to such a conception (Thompson 2008, p. 68). “Aristotelian categoricals” are claims about general aspects of the form of life the organism in question bears and what internal processes, traits, and behaviors are necessary conditions of the possibility that it unfolds.

Considering Mayr’s example of bird migration from this perspective once again helps to show how starkly Thompson’s Neo-Aristotelian view of teleology differs from the variety of Darwinian theorists mentioned in the first section. Recall that when asked why the warbler on Mayr’s porch migrates south at the end of the summer, Mayr answered that it is because ancestors with the same genetic make-up that “programs” migratory behavior given a certain seasonal change in temperature successfully reproduced and the warblers that one is presently observing have inherited these traits. When asked why warblers migrate south at the end of the summer, a Neo-Aristotelian response would be simply that they do so in order to reach a climate with more insects to eat. By calling the movement of the warbler “migration” we are already mobilizing a form of judgment that considers it a “vital” activity that serves a purpose within the organism’s unfolding life-cycle (See McLaughlin 2001 for a defense of teleology grounded in the features of self-sustaining, self-replicating systems, of which organic life-cycles are a paradigmatic example). This “first-order” identification and description cannot be rendered into “non-teleological” facts about phylogenetic history, ontogenetic development, genetic “programming” or the temperature changes that might induce the movement of limbs involved in the southward journey.

Philippa Foot elegantly summarizes the Neo-Aristotelian view of teleology in her book *Natural Goodness*. There, she offers a word of caution intended to preempt the objection that this explicit purposive language requires any metaphysically worrisome assumptions that the activity so described is intentional. She writes:

[T]he male peacock displays his brilliant tail in order to attract a female during mating season. The display serves this purpose. Let us call such language purposive language. But be careful here! Where something that S’s do is, in this sense, purposive we should beware of slipping over into saying of an individual S that it has this purpose when it does this thing. Plants grow upwards in order to get to the light, but it is fanciful to say that that is what my honeysuckle is trying to do or that is ‘its end.’ Migrating birds flying off in order to reach the southern insects do not have this end or purpose even though it could be said to be the end or purpose of the operation. (Foot 2003, p. 31)

Speaking of living activity as purposive in the Neo-Aristotelian sense (and in the Neo-Darwinian sense) does not require the assumption of mental capacities, intentions, or cognition in any plant, animal, insect, or cell. But, it does require recognition of the peculiar relations of causal dependence that link an organism’s activities to past, present, and future phases of the pattern of living activity that it bears. An organism displays a functional unity of interdependence between its parts and the whole, between its individual actions here and now, and its past and future. Foot’s semantic correction is important: Organisms as such do not *have* purposes or “ends,” but their traits and behaviors *serve* purposes. This functional unity and identity over time of an organism’s unfolding life-cycle is the context in which traits or behaviors can contribute to purposive vital activities at all, and through which these traits and behaviors can first be identified as the specific vital activities that they are.

Foot also specifically addresses the relation of this Neo-Aristotelian account of purposive language in biology to evolutionary theory in a manner that deserves full quotation. She writes: 

The history of a species is not, however, the subject with which Aristotelian categoricals deals. Their truth is truth about a species at a given historical time, and it is only the relative stability of at least the most general features of the different species of living things that makes these propositions possible at all. They tell of how a kind of animal, considered at a particular time and in its natural habitat, develops, sustains itself, defends itself, and reproduces. It is only insofar as ‘stills’ can be made from the moving picture of the evolution of species that we can have a natural history account of the life of a particular kind of living thing. And it is only insofar as we have a ‘natural history account’ that we can have a ‘vital description’ of individuals here and now. (Foot 2003, p. 29)

For both Thompson and Foot, describing the causal unfolding of life-cycle patterns is a task for biologists that is distinct from the task of understanding the natural-historical emergence, patterns of change, and even the underlying molecular “mechanisms” that have made such forms possible. Indeed, both Thompson and Foot rightly argue that the vital description of biological entities—describing what they *are*, how their parts “hang together,” and what they do—must antecede any question of origins, whether natural or supernatural, because it tell us what it is that we are explaining in the first place (Foot 2003, 32). In this way, teleological language is de-coupled from evolutionary history, but also from natural theology. Teleological language need not be used in answer to the question of origin or history, that is, of where living things came from. Instead, it captures the peculiar relations of interdependence that exist among whole organisms, their parts, and their behaviors that go on to shape the particular pattern of life that unfolds. In other words, it captures something about what an organism *is* as an unfolding and unified pattern of activity in nature.

This analytical separation of evolutionary questions from what we might call “evolutionary history” questions in biology makes a crucial methodological point, and contrasting them helps us appreciate is that there are two different things to be explained. One is internal to the life-cycle of the organism and it is about what is involved in representing an organism. The other is about how that organism itself came into being. Before one can explain the “evolution of” any traits, capacities, or behaviors, or even of organisms themselves, these *explananda* must first be brought into view. But vital descriptions involve imputing purposes to behaviors, traits, and even underlying molecular processes. Both Thompson and Foot insist that even accurately identifying the most basic behaviors of individual organisms—whether “eating,” “migrating,” “displaying,” “foraging,” or even the much more loaded language in social behavior of “cooperating,” “punishing,” “teaching”—already involves purposive language (Thompson 2008, p. 54; Foot 2003, p. 36). The philosophical concern to justify teleological judgments of purposiveness thus does not come from outside of biology or from any “external” and necessarily metaphysical interest, but rather from the need to give an account of the status of the sort of purposive language that we find to be indispensable for identifying and representing *biological* or *living* phenomena in the first place (Thompson 2008, p. 47).

Both Thompson and Foot thus carve out a unique view of purposiveness compared to those surveyed in the first section. On the one hand, like the mathematical critics of general design-principles such as “fitness” and “inclusive fitness” that offer a general, so-called “ultimate” purpose to capture the functional unity and model the behavior of organisms, Neo-Aristotelians argue that the purposive nature of an organism and its behavior does not require positing a single ultimate goal. Purposive descriptions and explanations that refer to “vital activities” within the context of an unfolding life-cycle pattern are ultimate in that they sufficiently answer “why” questions without the need to reference further purposes, but they do not require us to conceive of natural purpose in terms of a single, unified goal or objective that all organisms, and all their traits and behaviours, are directed towards. On the other hand, unlike these critics, Neo-Aristotelians argue that the purposive nature of biological activity is *constitutive* of our representation of life, of what living things are doing, and what is going on inside of them—to get rid of this language is to fail to bring *biological* reality into view (Thompson 2008). For this reason, the wider context of the life-cycle pattern of whole organisms maintains a distinct priority in the representation of life.

While the Neo-Aristotelian writers surveyed above maintain realist views of the purposive character of life, this does not appear to be necessary. One can maintain an *error theory* or a *methodological teleologist* account of “vital descriptions” that impute a teleological character to an organism and its activities. However, Neo-Aristotelian accounts of the basis of teleological language and its role in making sense of the biological world insist that these anti-realist accounts are tantamount to rejecting the reality of living processes. This insistence is certainly behind the tendency of Neo-Aristotelian thinkers to be realists concerning teleology and behind their motivation to reject *error theory* or *methodological teleology* accounts. But while it is important to note that rejecting the reality of life is involved in adopting a non-realist approach to teleology, it is also important to recognize that non-realist interpretations of teleological language do not appear to be incompatible with Neo-Aristotelian perspectives on the theoretical basis and role of this language in the representation of life. We might still maintain that although distinctly *living* processes require teleological concepts to even be represented, the mind-independent world does not indeed contain such processes.

## Neo-Darwinism and Neo-Aristotelianism: questioning the purpose of reproduction

The *Darwinian teleological realist* will no doubt object at this point that this cannot be where the story ends; there is a reason why reproduction is considered the event whereby an organism fulfills its *raison d’être* in evolutionary thinking. The *Neo*-*Aristotelian teleological realist* argues that the foundational license for viewing even the simplest organisms as purposive lies in the peculiar causal interdependence of phases and processes in a life-cycle pattern that teleological judgments pick out. But an evolutionist will insist that life-cycle patterns (and of course the variety of life-cycles organisms go through) are not simply ready-made, they are shaped by selective, evolutionary pressures. The first section showed that the *Darwinian teleological realist* justifies purposive language by regarding the process of evolution as yielding whole organisms that are organized so as to allow them to spread their genetically controlled and heritable traits efficiently and abundantly. Indeed, on the basis of this framework, scientists working in the area of life-history evolution argue that life-cycle patterns of maturation, growth, age of reproduction, and senescence can be demonstrated to optimize between specific trade-offs forced on organisms by biological, social, and ecological conditions and constraints (Stearns 1992). Discovering life-cycle patterns, traits, and behaviors that optimize various aspects of life-cycle necessary for reproductive success in tandem with biological and environmental constraints is taken as evidence for the Darwinian view that natural selection shapes organisms with the sole ultimate objective of spreading their genes (Krebs, Davies, and West 2012).

If such a Darwinian rejoinder is committed to an *error theory* with regard to teleology and so to this notion of an ultimate objective, then it is surely incompatible with *Neo*-*Aristotelian teleological realism*. But if the notion of an ultimate evolutionary objective is interpreted in terms of *methodological teleology*, then it is not in principle incompatible with *Neo*-*Aristotelian teleological realism*. What this means is that Neo-Aristotelian realists can accept the concept of an ultimate goal as merely a formalized heuristic tool that helps scientists investigate and model behavior, model evolutionary outcomes, and reveal how complex behavioral repertoires emerge and interact to determine such outcomes. At the same time, they can insist that organisms are still genuinely purposive entities, but representation of their purposive nature is not grounded in and does not require the notion of an ultimate goal. When it comes to contrast between the *Darwinian teleological realist* and the *Neo*-*Aristotelian realist* views, the conflict is much more pronounced.

The first thing to recognize is that the differences between these two realist views are not just semantic; they pick out different orders of purposiveness that are justified by different conceptions of the purposive nature of biological entities. The key point here is that in raising this form of the question of “ultimate” purpose, the *Darwinian teleological realist* too presses us to seek and to provide an explanation of biological traits and behaviors through reference to an order of purposes. But it is unique in that it presses us to identify one purpose as the “ultimate” or “final” purpose *for the sake of which* all other vital activities occur. The “ultimate” objective of inclusive fitness offers a view about the teleological order of purposes that applies to all vital activity; an individual organism simply *is* an inclusive fitness-seeking entity whose objective is the spread of its genes, whether *directly* through its own reproduction or *indirectly* through the success of those who share its genes. All other vital activities are subordinate and in service to this “ultimate” objective. The power of this view is the way it orients rigorous behavioral observation, makes predictions about how living things will behave under various circumstances, and shows unity to the game of life as it is played out in the countless variety of life-forms. Moreover, this aspect of the Darwinian view underwrites the widespread claim, explicitly found in writings by Daniel Dennett, that evolutionary biology offers a so-called “ultimate” explanation of purpose in life (Dennett 1995).

The problem with this Darwinian view can be seen, I suggest, by focusing on how this view leads us to understand the process of reproduction. The attraction of the Darwinian “ultimate” purpose is obvious. It answers an apparent regress of purposes by identifying an objective for the organism as a whole and for it’s entire life-cycle pattern. As Foot wrote, “the male peacock displays his brilliant tail in order to attract a female during mating season. The display serves this purpose.” But it of course natural to ask what purpose attracting a female serves? What purposive *rationale* is there for reproduction? One appealing feature of Richard Dawkins’ classic and influential distinction between *replicators* and *vehicles* was that it gives an answer to this question (Dawkins 1976). The Darwinian “final end” too satisfies our cognitive need for a regress stopper in the form of a rationale, a “final” rationale for what an organism is, how it is, and what it does. An organism simply is a temporary “vehicle” for replicators that program heritable traits and reproduction serves the purpose of spreading them. The “gene-centered” view here comes to be favored not only because it provides a sought after account of how evolutionary change by natural selection works, but also because it offers an “ultimate” purpose and rationale. Ironically, while Dawkins appears to be critical of and to get rid of teleological explanation in biology, it is reintroduced at this crucial point.

Of course, the evolutionist need not understand reproduction as purposive in this way. The right way for the *Darwinian teleological realist* to make her point is to resist the temptation to suggest that reproduction serves a purpose (whether of genes, individuals, groups, or species) while stressing that all other life-cycle events, vital processes, and behaviors serve the purpose of reproduction. This way of putting the matter recognizes that we do need not answer the question of what purpose reproduction serves—i.e., “why” do organisms reproduce? Yet we are right to seek purposive explanations of an organisms traits and one might still claim that all behaviors, traits, and phases of an organism’s life cycle serve the sole, ultimate objective of successful reproduction. Thus, while reproduction itself does not serve a purpose, other traits and behaviors serve the purpose of reproduction. This preserves the “ultimacy” of reproduction in the range of an organism’s activities while resisting the temptation to make claims about the *raison d’etre* of an organism.

However, here we must question whether reproduction can be accorded the status of “ultimacy” and “finality” that the *Darwinian teleological realist* view gives it. Nothing about the reproductive phase of the life-cycle forces us to consider it the vital activity *for the sake of which* all other vital activities occur. Indeed, it is not clear that biology requires us to posit a universal purpose of all living activity or to stop the regress of “why” questions by introducing a single, “ultimate” purpose of life. Warblers migrate southward from New Hampshire in August in order to find insects to eat—full stop. There is no need for further purposive explanation of this activity. This is likely to strike the *Darwinian teleological realist* as a mistake because the crucial difference that an evolutionary point of view makes to an understanding of natural purpose is precisely to highlight the priority of reproduction in the spectrum of an organism’s goals. But the *Neo*-*Aristotelian* view helps us see that relating an organism’s traits and behaviors to any of its “vital activities” constitutes a sufficient purposive explanation.

Another way to put this point is to think about the different values placed on survival and reproduction in evolutionary thought. Because reproductive success is the criterion of natural selection and a necessary condition of evolution, thinking about the process of evolution tempts us to see reproduction as the “ultimate” aim of the individual organism. It also tempts us to subordinate the organism as a privileged “unit” of biological reality and to see it as merely a “vehicle” for units that persist beyond it. But the explanatory pressure to supply an “ultimate” goal asks for one rationale too many. When thinking not of evolutionary change but instead of life-cycle patterns of individuals and what is required for them to unfold, the “ultimacy” of reproduction recedes. Organisms do all sorts of things in the course of their life-cycles, and the functions of many of their activities can be identified without referring to reproduction. Indeed, organisms can and do function even if successful reproduction does not take place (Thompson 2008, p. 68).

If we grant that there is a flaw in the general claim that reproduction serves a purpose and in the more specific claim of the *Darwinian teleological realist* that reproduction has the status of “ultimacy” and “finality” in terms of a characterization and explanation of an organism’s traits, there still remains an important point that the Darwinian view makes to complement the Neo-Aristotelian account of natural purposiveness. This relates to the dynamic of competition that was so central to the Darwinian understanding of evolution and that Darwin originally drew from Thomas Malthus’s writings on population dynamics. As populations increase through continuous reproductive success, inter-specific competition would ensue between different “forms of life” and intra-specific competition would ensue for limited food resources and reproductive opportunities. Empirical field observation by behavioral ecologists has shown that competition for resources and mating opportunities is crucial for understanding the purposes that social behaviors come to serve in the life-histories of organisms (See, for example, Clutton-Brock and Harvey 1995). These dynamics give behavioral, morphological, and physiological traits distinctively competitive and cooperative purposes within these unfolding life-cycle patterns.

Neo-Aristotelian conceptions of the teleological organization of individual organisms can profit from Darwin’s understanding of evolution by incorporating the view of the living world as an arena in which cooperation and competition takes place. For instance, aggressive behavior in a female meerkat can often serve the purpose of protecting a valuable resource or guarding a mate from rivals or it can help to establish an immigrant male meerkat in a new group by fending off resident males (See Lott 2012 for an extensive and insightful discussion of these issues in relation to moral theory). A “vital description” of life-history patterns of Damaraland mole-rats would surely involve, for example, the fact that the female “queens” of colonies live 25 years longer than “subordinate” females, many of whom never reproduce successfully and live much shorter lives. This is because typical female mole-rat life-cycles involve competition for mating opportunities with unrelated males, and male mole rat life-cycles involve competition for the chance to mate with the queen. It is examples like these that contributed to the appeal of Dawkins’ original presentation of the “selfish” objectives of evolutionary “vehicles” and any Neo-Aristotelian account of purposiveness must recognize these competitive purposes.

Nonetheless, even here we must be careful to observe that teaming up with others in the struggle for life is just as prevalent a strategy for meeting vital needs as competitive struggle, which gives rise to distinctly cooperative purposes as well (Gardner 2008; Nowak 2011; Kropotkin 1972/1902). The Neo-Darwinian view fills out the Neo-Aristotelian framework for understanding natural purpose by intensifying our sense for the competitive and cooperative dynamics of social evolution in light of which “vital activities” need to be understood and identified (Queller and Strassmann 2009). Another important aspect of the social existence of organisms, which we briefly encountered in the first section, is that it offers examples of cases whereby an individual organism’s reproductive phase and life-cycle *as a whole* can indeed come to “serve a purpose” beyond itself. This is the case in the examples of symbiosis studied in the relationship between an organism and its microbiome and in so-called “superorganisms” such as some insect colonies or multi-cellular organisms in which individual reproduction is suppressed and individual life-cycles themselves play a role in the vital activities of a higher-order and functionally unified whole. In these biological systems, whole organisms themselves can be said to serve the purpose of reproduction, but in these cases it is of the reproduction of the higher-order whole organisms and collectives to which they belong. Of course, while in these cases whole organisms can be said to serve the purpose of reproduction, the reproduction of these higher-order entities does not serve any available vital purpose.

In this section, I have argues that it is misguided to see reproduction as serving the purpose of spreading genes or traits, it is also misguided to see reproduction as serving the purpose of outcompeting other organisms, and it is misguided to see reproduction as serving the purpose of evolution as a whole—unless of course one resorts to a form of theism. The temptation to say things like this is again the sense that evolutionary biology must, or can, give an answer to the question of what purpose reproduction serves, of “why” living things reproduce. But for biology, reproduction must remain a given, “brute” fact. Reproduction serves the purpose of reproduction; in other words, reproduction is one “vital activity” among others in the Thompson and Foot sense. But since this is neither informative nor explanatory, it appears that the question of the purpose of reproduction is not a biologically meaningful one. Just like Aristotle proposed that living things reproduce in order to imitate the perfection of the immortal heavenly bodies, biologists and philosophers today may risk to venture into their own “myth-making” when trying to give an answer to the question of the “ultimate” purpose of an organism’s life-cycle. The temptation to propose that evolutionary biology offers a purposive “ultimate” explanation of life, so often succumbed to by self proclaimed “evolutionists” like Daniel Dennett, or that it says something about the “ultimate” purpose of living things in nature, is a temptation to exceed what biology, and natural science in general, can and need offer (Dennett 1995).

## Conclusion

Mayr’s (1961) paper posed a fascinating problem that continues to linger in evolutionary biology and the philosophy of biology and I have tried in this paper to draw out many of its wider implications—some of them even venturing into questions that might more accurately be called theological. How can we make sense of the pervasiveness and apparent indispensability of the language of purpose next to other kinds of cause and effect in the biological world? Such language is rife in both scientific literature on evolution and in popular accounts of living things. In this paper, I have reconstructed different paths in the scientific and philosophical literature for understanding this language: *teleological error theory*, *methodological teleology*, and finally both *Darwinian* and *Neo*-*Aristotelian teleological realism*. *Teleological error theory* and *methodological teleology* reject the reality of purposive characteristics of the living activity of whole organisms or their parts altogether. The *Darwinian teleological realist* argues that any teleological language must be understood in relation to an “ultimate” objective of reproduction or that is derived from how evolution by natural selection shapes organisms and their behavior. Its focus on mechanisms of causation in evolution leads it to concentrate on genes and heritable traits as the fundamental “units” of biological reality. The *Neo*-*Aristotelian* argues that purposiveness picks out the peculiar causal inter-dependence of living activity within the wider context of a life-cycle pattern and the “vital activities” that sustain it. Its “non-evolutionary” focus leads it to view the organism as the fundamental unit of biological reality. Both *Darwinian realist* and *Neo*-*Aristotelian* accounts reject “deflationary” accounts, such as Mayr’s, that translate questions of purpose into questions of genetic and physiological causation, or phylogenetic history. But the Neo-Aristotelian argues that there is a purposive “cause,” or equivalently, a purposive “because…” built into representing anything as living.^1^

One powerful reason to desire such a realist vindication of teleological language is an uncontroversial fact that I alluded to earlier. Some products of evolution are purposive and agential, namely ourselves and other “cognitively complex” animals. It is a desideratum to understand how the teleological characteristics of psychologically directed agency relate to the characteristics of biological life more generally. The realist accounts of teleology surveyed here both support conceptions of cognitively guided purposiveness as pre-figured by the characteristics of non-cognitive life more generally, but in very different ways. If the organism *really does* have an ultimate objective of reproduction, then we are in a position to speak not only of what an organism does, but also of what it *should* do given that it has this natural purpose. Both the Neo-Aristotelian and Darwinian realist positions can be the source of the peculiar way in which distinctly normative “ought” claims can be thought to arise out of an understanding of biology and evolution, and this is the reason for some of the checkered history of evolutionary thought in realms of ethics and political theory. The validity of all normative and evaluative judgments derived from the conception of “fitness” in the behavioral sciences rests on a realist view of natural purpose. One motivation for denying realism, then, can come from the sense that this sort of normative talk is value-laden and therefore unscientific and metaphorical. Another can come from the side of normative ethical theory through the judgment that the sorts of norms evolutionary biology might appear to entail are not in line with our best moral reasoning. The Neo-Aristotelian views surveyed here are also aimed at offering resources for a *rapprochement* between biology and normative moral theory, but one that does not require thinking in terms of reproductive success (Thompson 2008; Foot 2003; Lott 2012).The question of the place and status of teleology in biology in general is foundational for understanding the relationship between biology and normative ethical theory, and I hope to have shown here a variety of different positions that might be relied upon in order to address such questions.

Finally, I have offered considerations in favor of the *Neo*-*Aristotelian* position that the question of whether or not and in what ways biological entities are purposive is not an evolutionary question of their origins and how they change over time. Nor does this question force us to first answer questions of how living things arose from non-living matter in the first place. Indeed, only when we have decided whether or not, and in what sense, organisms really are purposive does it make sense to ask how natural purposes could have arisen out of non-purposive material processes at all. Of course, the power of Darwinian theory is its usefulness in conceiving and answering “origin” questions, and the general cumulative nature of evolutionary change orients research into how any of the natural purposiveness we observe arose in the first place. But the question of origins of life presupposes a representation of what living things *are* and this is something that evolutionary theory does not provide. Furthermore, just as evolutionary theory need not and cannot give a purposive explanation of “why” organisms reproduce, it also cannot give an “ultimate,” purposive explanation of “why” life evolved out of non-living matter. These questions pertain to biological reality, but they are not the kinds of questions that are tractable for evolutionary, or any other form of biological explanation.

## Footnotes

1 Thanks to Micah Lott for this formulation.
